# Comparative genomic analysis of esophageal squamous cell carcinoma between Asian and Caucasian patient populations

**DOI:** 10.1038/s41467-017-01730-x

**Published:** 2017-11-16

**Authors:** Jiaying Deng, Hu Chen, Daizhan Zhou, Junhua Zhang, Yun Chen, Qi Liu, Dashan Ai, Hanting Zhu, Li Chu, Wenjia Ren, Xiaofei Zhang, Yi Xia, Menghong Sun, Huiwen Zhang, Jun Li, Xinxin Peng, Liang Li, Leng Han, Hui Lin, Xiujun Cai, Jiaqing Xiang, Shufeng Chen, Yihua Sun, Yawei Zhang, Jie Zhang, Haiquan Chen, Shijian Zhang, Yi Zhao, Yun Liu, Han Liang, Kuaile Zhao

**Affiliations:** 1 Department of Radiation Oncology, Fudan University Shanghai Cancer Center, Shanghai, 200032 China; 2 Department of Oncology, Shanghai Medical College, Fudan University, Shanghai, 200032 China; 3 Graduate Program in Quantitative and Computational Biosciences, Baylor College of Medicine, Houston, TX 77030 USA; 4 Department of Bioinformatics and Computational Biology, The University of Texas MD Anderson Cancer Center, Houston, TX 77030 USA; 5 Department of General Surgery, Sir Run Run Shaw Hospital and Institute of Translational Medicine, Zhejiang University School of Medicine, Hangzhou, 310000 China; 6 Innovation Center for Minimally Invasive Technique and Device, Zhejiang University, Hangzhou, 31000 China; 7 Department of Pathology, Fudan University Shanghai Cancer Center, Shanghai, 200032 China; 8 Department of Biochemistry and Molecular Biology, The University of Texas Health Science Center at Houston McGovern Medical School, Houston, TX 77030 USA; 9 Department of Biostatistics, The University of Texas MD Anderson Cancer Center, Houston, TX 77030 USA; 10 Department of Thoracic Surgery, Fudan University Shanghai Cancer Center, Shanghai, 200032 China; 11 Precision Scientific (Beijing) Co., Ltd, Beijing, 100085 China; 12 Institute of Biomedical Sciences, Fudan University, Shanghai, 200032 China; 13 Department of Systems Biology, The University of Texas MD Anderson Cancer Center, Houston, TX 77030 USA

## Abstract

Esophageal squamous cell carcinoma is a major histological type of esophageal cancer, with distinct incidence and survival patterns among races. Although previous studies have characterized somatic mutations in this disease, a rigorous comparison between different patient populations has not been conducted. Here we sequence the samples of 316 Chinese patients, combine them with those from The Cancer Genome Atlas, and perform a comparative analysis between Asian and Caucasian patients. We find that mutated *CSMD3* is associated with better prognosis in Asian patients. Applying a robust computational strategy that adjusts for both technical and biological confounding factors, we find that *TP53*, *EP300*, and *NFE2L2* show higher mutational frequencies in Asian patients. Moreover, *NFE2L2* mutations correlate with the allele status of a nearby high-*F*st SNP, suggesting their potential interaction. Our study provides insights into the molecular basis underlying the striking racial disparities of this disease, and represents a general computational framework for such a cross-population comparison.

## Abstract (web summary)

Esophageal squamous cell carcinoma (ESCC) exhibits differences in incidence and survival patterns among races. Here, analysis of Chinese and TCGA ESCC patients reveals that Asian patients exhibit higher TP53, EP300 and NFE2L2 mutational frequencies, and mutated CSMD3 associates with better prognosis.

## Introduction

Esophageal cancer disproportionately affects certain ethnic groups and races. More than half of global esophageal cancer cases occur in China, with 477.9 thousand new cases diagnosed in 2015^1^; whereas in the United States, the incidence of esophageal cancer is much lower, with 17.9 thousand new cases diagnosed in 2016^2^. Histologically, esophageal squamous cell carcinoma (ESCC) is the major type in Asian populations (e.g., 90% of Chinese patients); while esophageal adenocarcinoma is the dominant type in western countries^3^. Among American patients, the incidence rate for ESCC is 81% higher among Asian/Pacific Islander males compared with the rate for white males, but is 64% lower than the rate for black males^4^. Furthermore, there are substantial differences among races in terms of cancer survival^5,6^.

Besides external factors such as environmental differences, behaviors, and treatment options^7^, it is essential to investigate the molecular basis underlying the striking racial disparities in ESCC. Several recent studies^8–12^ have characterized significantly mutated genes in this disease, but a rigorous cross-population comparative analysis remains challenging for two notable reasons. First, mutation detection is largely affected by technical factors, including the sequencing platform, mapping pipeline, sequencing depths, and mutational calling algorithms, all of which should be carefully controlled in such an analysis. The effects of these factors are often large for mutation data collected from different studies^13^. Second, biological factors such as patient age, gender, and clinical stage also strongly impact the mutational status of a gene^14,15^, and these confounding effects should be considered when detecting race-specific mutational differences.

## Results

### Overall analytic strategy for cross-population comparison

**Fig. 1:**
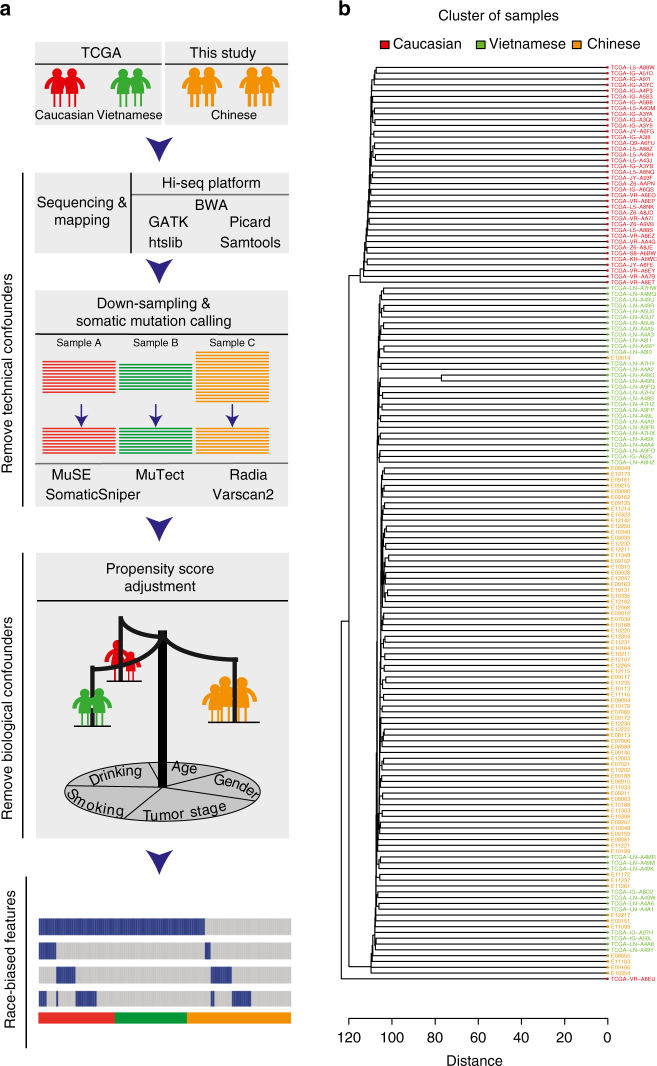
Schematic representation of the analytic strategy. **a** ESCC whole-exome sequencing data of three patient cohorts, Caucasian, Vietnamese and Chinese, were respectively obtained from this study and TCGA. Our strategy includes two major steps to remove confounders. To remove technical confounders, we applied the same procedure to process sequencing reads generated from the Hi-seq sequencing platform. We then performed downsampling to balance the depth of coverage among the three cohorts, followed by a stringent method to call somatic single-nucleotide mutations using multiple mutational callers. Second, to remove biological confounders, we calculated propensity scores, reweighted samples in the cohorts, and compared gene mutation frequencies between two balanced cohorts. We considered five biological factors (age at diagnosis, gender, tumor stage, smoking history, and alcohol consumption history) in the propensity score adjustment. **b** Hierarchical clustering pattern of patient samples by common SNP status in the exonic regions. Asian patients and Caucasian patients form two distinct clusters

To overcome these challenges, we developed a robust computational strategy to detect race-biased mutated genes in ESCC between Asian patients (Chinese patients characterized by this study and Vietnamese patients characterized by the recent The Cancer Genome Atlas (TCGA) study^8^) and Caucasian patients (characterized by the TCGA) (Fig. 1a). Briefly, we employed the Illumina sequencing platform to generate whole-exome sequencing (WES) data for Chinese patients and applied the same processing pipeline to generate mapping files for both Chinese and TCGA patients. Then, we implemented a down-sampling strategy to remove the batch effects due to the sequencing depth variation between the studies and robustly detected mutations using the same strategy of multiple mutation callers. Finally, we employed the propensity score analysis^16^ to remove the confounding effects due to biological factors.

To conduct the cross-population comparison, we performed WES for 78 Chinese patients with ESCC (Supplementary Data 1). This patient cohort was combined with Caucasian (*n* = 39) and Vietnamese (*n* = 41) patients with ESCC in TCGA as a discovery cohort to identify race-specific mutational features in a genome-wide, unbiased way. Among the three populations surveyed, Vietnamese and Chinese populations are geographically close and belong to the same Mongolian race. We first examined the genetic relationship of the patients based on common single-nucleotide polymorphism (SNP) status of the 158 WES samples. The hierarchical clustering identified two major clusters (Fig. 1b): one cluster contained mixed Chinese and Vietnamese patients, and the other consisted of Caucasian patients only. In addition, alcohol intake is one important factor for ESCC development, especially in Asian population^17^. We therefore compared germline variations of two important alcohol metabolizing enzymes, *ALDH2* (rs671) and *ADH1B* (rs1229984) in the three populations and found very similar patterns between Chinese and Vietnamese patients (Supplementary Fig. 1). These results confirmed the genetic similarity between Chinese and Vietnamese patients, justifying that they should be combined as an Asian cohort in the comparison. We further characterized another Chinese cohort of 313 patients (of which 75 had been characterized with WES) using targeted sequencing (283 select genes)^18^ as a validation cohort to confirm the WES findings (Table 1, Supplementary Data 1). We applied the same analytic pipeline (both mapping and mutation calling) to the WES data of Chinese patients and TCGA patients. We validated our mutation calls in two ways. First, for TCGA patients, compared to the latest refined TCGA mutation data, 96.4% of our non-silent mutation (missense mutation, nonsense mutation and non-stop mutation) calls on the same patients were reported. Conversely, 85.1% of TCGA mutation calls were confirmed in our analysis. Second, for the Chinese patients in the discovery cohort, 95.3% of our mutations (564/592) detected in WES were validated with independently targeted sequencing in the same patients. Further, the variant allele frequencies characterized by WES and targeted sequencing were highly correlated (*r* = 0.91, Supplementary Fig. 2). These results demonstrate the high quality of our mutation data.

**Table 1:** Characteristics of patient cohorts surveyed in this study

Clinical factors	TCGA		This study	
	WES		WES	Additional targeted sequencing
	Caucasian	Vietnamese	Chinese	Chinese
*Gender*				
Male	29	39	65	215
Female	10	2	13	23
*Age (years)*				
30–40	0	2	0	1
40–50	6	11	11	27
50–60	19	15	35	106
60–70	9	9	27	100
70–80	4	4	5	4
80–90	4	0	0	0
*Tumor stage*				
I	6	0	0	12
II	17	31	63	118
III	14	9	15	108
IV	2	1	0	0
*Smoking history*				
Smoked/smoking	25	22	46	160
Never	11	19	32	78
Unknown	3	0	0	0
*Alcohol history*				
Yes	25	30	36	105
No	12	11	42	133
Unknown	2	0	0	0

### Prognostic power of *CSMD3* mutation status in Asian patients

**Fig. 2:**
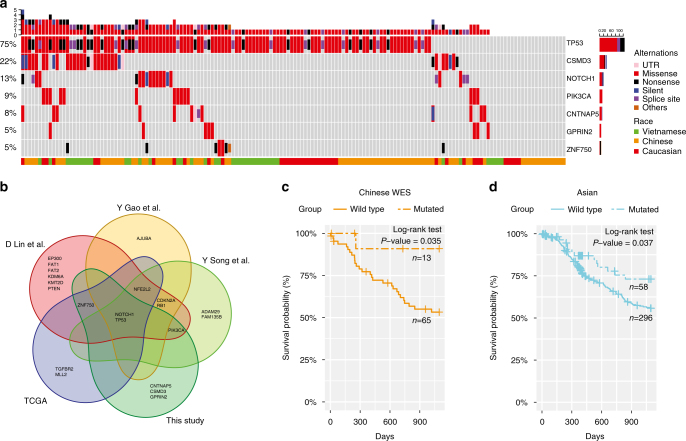
Significantly mutated genes in ESCC. **a** Significantly mutated genes (SMGs) identified by MutSigCV on a combined cohort of Caucasian, Vietnamese, and Chinese WES samples. Each column denotes an ESCC patient, and each row is a gene. On top is the number of somatic mutations per sample. On the left are the mutation frequencies of each SMG. The bar plot on the right shows the composition of mutations in the gene. Genes are ordered by their mutation frequencies. **b** Overlap of SMGs reported by five studies. **c**, **d** Kaplan–Meier curves according to the mutational status of *CSMD3* gene in **c** 78 Chinese WES cases and **d** other 354 Asian cases (consisting of 41 Vietnamese WES cases and 313 Chinese targeted sequencing cases). Mutated groups show significantly better overall survival outcomes (log-rank test)

Combining the WES mutation data from 158 Chinese, Caucasian and Vietnamese patients, we detected seven significantly mutated genes using MutSigCV^19^ (*q*-value = 0.1) with a mutation frequency of ≥5% (Fig. 2a). Among them, *TP53*, *NOTCH1*, *PIK3CA*, and *ZNF750* have been reported in previous ESCC sequencing studies^8–11^. Among novel significantly mutated genes identified, the function of *CSMD3* (CUB and Sushi multiple domains protein 3) remains largely unknown in this disease (Fig. 2b). *CSMD3* inhibition has been reported to affect the proliferation of airway epithelial cells^20^. Since this gene contains very long introns, its elevated mutation rate might be due to mutational heterogeneity (although MutSigCV has corrected for gene-specific background mutation rates^19^). We therefore examined the correlation of *CSMD3* mutation status with patient survival times, which is orthogonal to the mutation rate analysis. We found that among Chinese patients with WES data (*n* = 78), patients with mutated *CSMD3* showed significantly better survival time than those with the wild-type allele (Fig. 2c, log-rank *P* = 0.035), and we observed a similar pattern in Asian patient samples (*n* = 354) (Fig. 2d, log-rank *P* = 0.037). However, there was no such pattern in Caucasian patients (Supplementary Fig. 3). Intriguingly, a similar prognostic pattern of *CSMD3* has been recently reported in Chinese patients with lung squamous cell carcinoma^21^. These results suggest that the mutational status of *CSMD3* is a prognostic marker for Asian populations.

### Mutational rate comparison among patient populations

**Fig. 3:**
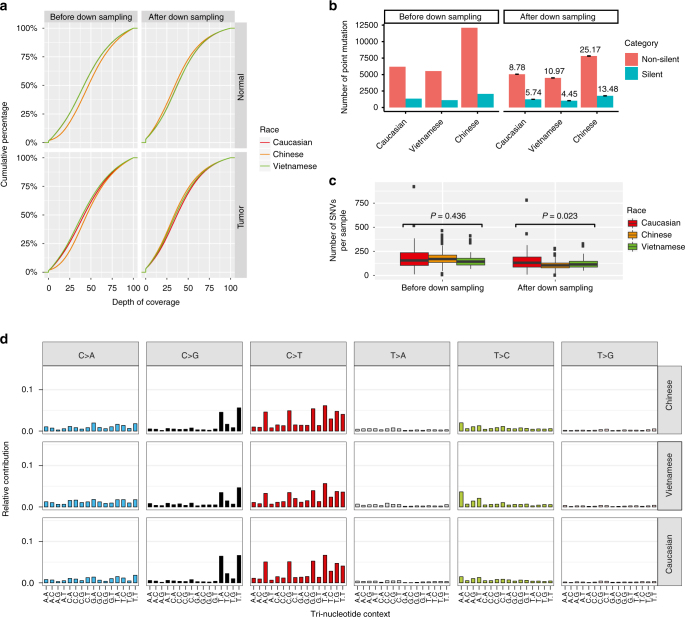
Comparison of mutational patterns of different ESCC patient populations. **a** Distribution of depth of coverage of exons captured in WES. Chinese tumors and matched normal tissues had higher depth of coverage than those of TCGA ESCC cases before downsampling. After downsampling, the three patient cohorts had similar distributions of depth of coverage. **b** The number of somatic mutations for each patient cohort before and after downsampling. The error bars and numbers on the top of the bars were calculated based on mutation calls from 10 downsampling iterations. **c** Box plots showing the distribution of the number of somatic mutations in each sample in the three cohorts before and after downsampling. The center lines in the boxes are the median numbers of somatic mutations for different patient cohorts, while the upper and lower hinges are the 25th and 75th percentiles. Whiskers above and below the boxes indicate 1.5 times interquartile range. Individual points are those outside of the range. **d** Mutational signature of each cohort after downsampling

To rigorously compare the mutational rates among the ESCC populations, we first assessed the sequencing depth of exons and found that samples from Chinese patients had a considerably greater depth than those from Caucasian or Vietnamese patients (Fig. 3a), which would result in an overestimation of the mutation rate in Chinese patients. We therefore implemented a random read downsampling strategy so that each exon had similar sequencing depths across the three patient populations. We performed downsampling for 10 times and called somatic mutations independently in order to assess random effects. As a result, the total mutation numbers were respectively reduced by 16.5–17.1% and 16.1–16.6% in Caucasian and Vietnamese patients; while the mutation number was reduced by 31.9–32.7% in Chinese patients due to a larger down-sampling effect (Fig. 3b). We observed very consistent somatic mutation callings across different downsampling iterations (Supplementary Fig. 4), and therefore used consensus mutation calls (in ≥8 times) for further analyses. After downsampling, Chinese and Vietnamese patients showed a significantly lower mutation rate than Caucasian patients (ANOVA, *P* = 0.023); whereas before downsampling, there was no difference among these three populations (ANOVA, *P* = 0.44) (Fig. 3c). This result highlights the importance of controlling for technical confounding factors in such a comparison. We also examined the mutational signatures and found very similar patterns across the populations (Fig. 3d).

### Identification of race-biased mutated genes

**Fig. 4:**
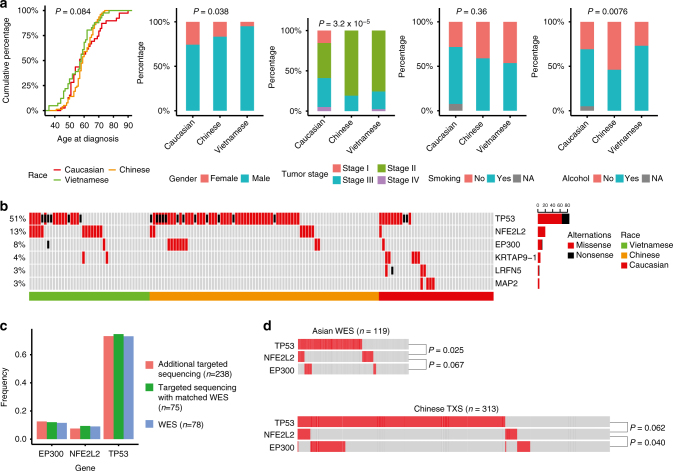
Race-biased genes identified by the propensity score algorithm. **a** Distribution of five biological factors (age at diagnosis, gender, tumor stage, smoking history, and alcohol consumption history) in three patient groups, justifying a need to balance these confounders. Analysis of variance test was used to calculate the *P*-value for age at diagnosis, and chi-squared test was used for the other factors. **b** Mutational landscape of six race-biased genes. Genes were ordered by mutational frequencies and samples were grouped by race groups. **c** Mutational frequencies of *EP300*, *NFE2L2*, and *TP53* in Chinese patients with WES, with targeted sequencing with matched WES and with additional targeted sequencing data. **d** Mutual exclusivity of *TP53*, *NFE2L2*, and *EP300*. On the top are based on the WES data of combined Chinese and Vietnamese patients, and on the bottom are the targeted sequencing data for 313 Chinese patients. *P*-values were calculated by CoMEt algorithm

To robustly identify the race-biased mutated genes, we assessed the distributions of several key biological/clinical factors in the three populations. We found that patient age at diagnosis, gender, tumor stage, and alcohol consumption all showed some significant bias across the populations (ANOVA for age, and chi-square test for gender, tumor stage, smoking, and alcohol consumption; Fig. 4a). We therefore employed propensity score analysis^16^ to adjust for the potential effects of these confounders. Importantly, samples with the same propensity score have the same distribution of measured confounders, so balancing the confounders can be achieved by simply balancing the propensity scores^22^. Using this algorithm, we identified six genes between Asian and Caucasian patients (false discovery rate (FDR) <0.1). Specifically, *TP53*, *NFE2L2*, and *EP300* showed a significantly higher mutation rate in Asian populations; while *KRTAP9-1*, *LRFN5*, and *MAP2* showed the opposite patterns (Fig. 4b). We further confirmed the high mutation frequency of *TP53*, *NFE2L2*, and *EP300* using targeted sequencing data on the discovery and validation cohorts (Fig. 4c). Interestingly, the mutational status of these three genes showed marginally significant mutual exclusivity (CoMEt algorithm, Fig. 4d), and analyses on additional patient cohorts are required to confirm this pattern.

### Correlation of somatic mutations with a 5′UTR SNP in *NFE2L2*

**Fig. 5:**
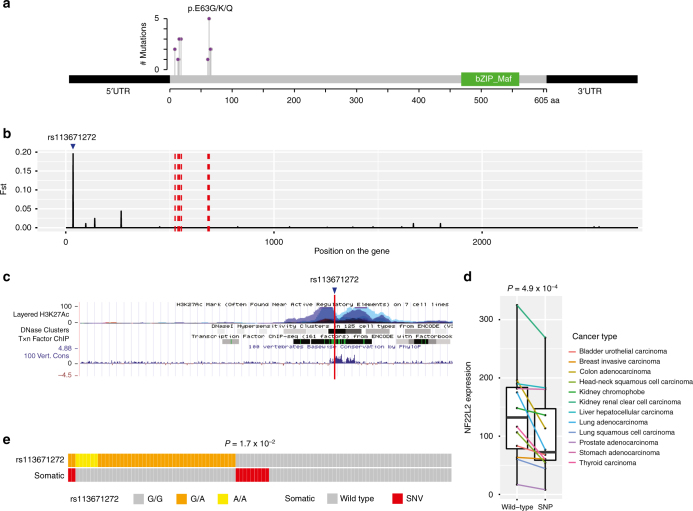
Correlation of *NFE2L2* mutations with a nearby high-*F*st SNP in Asian patients. **a** Mutational distribution on *NFE2L2* from all the WES samples. There are two major mutational hotspots. **b** *F*st-index values of the SNPs on the exonic regions of *NFE2L2*, among which rs113671272, located in 5′UTR, shows the highest *F*st index in the comparison between southern Han Chinese and European. **c** The gene regulation tracks from the UCSC genome browser show that rs113671272 is located within a region with high-density regulatory binding sites and high conservation scores. **d** The effect of the SNP rs113671272 on the mRNA expression level of *NFE2L2* across 12 TCGA cancer types. After excluding tumor samples with somatic mutations in *NFE2L2*, the cancer types with at least three samples in the SNP-containing group were included in the analysis. The expression levels between the sample groups (with or without the SNP) across cancer types were compared with paired Wilcoxon signed-rank test. **e** Mutual exclusivity pattern of rs113671272 SNP and the somatic mutation status in the Asian WES samples with sufficient coverage. *P*-value was calculated by CoMEt algorithm

We next examined the detailed mutational distributions in these genes. The *TP53* mutations are widespread throughout the whole gene, while the mutations in *EP300* are enriched in the domain of HAT_KAT11, as previously reported^10^ (Supplementary Fig. 5). *NFE2L2* (also known as *NRF2*) is of particular interest: this gene is a transcription factor that regulates many proteins involved in response to injury and inflammation as well as cellular defense against oxidative stress; *NFE2L2*-knockout mice are more susceptible to esophageal carcinogenesis than wild-type mice^23^; and its mutations have been recently reported to enrich in Vietnamese patients^8^. We identified several mutation hotspots at the first 100 amino acids of the protein encoded by *NFE2L2* and found that the most frequently mutated site resided in the coiled coil region (Fig. 5a). To examine whether the race-biased mutation pattern correlates with some germline signature of this gene, we calculated the fixation index^24^ (a commonly used measure of population differentiation due to genetic structure), *F*st for common SNPs of this gene by comparing European and Chinese populations using data from the 1000 Genomes Project^25^. We found a SNP (rs113671272) with a very high *F*st score in the 5′UTR of *NFE2L2* (Fig. 5b). This SNP is located in one region with strong DNaseI hypersensitivity and high-density regulatory binding sites, suggesting potential functional effects on the transcriptional regulation of *NFE2L2* (Fig. 5c). To further investigate the potential effects of this SNP on *NFE2L2* expression, we integrated the SNP data from International Cancer Genome Consortium (ICGC) whole-genome sequencing and TCGA RNA-seq data to compare the mRNA expression level of *NFE2L2* between cancer samples with and without this SNP and found that the presence of this SNP was associated with a significantly lower *NFE2L2* expression across cancer types (paired Wilcoxon signed rank test, *P* = 4.9 × 10^−4^, Fig. 5d **)**. Interestingly, the mutational status for Asian patients was strongly associated with the allele status of this SNP (CoMEt, *P* = 1.7 × 10^−2^, Fig. 5e). This intriguing pattern suggests their potentially interacting relationships.

## Discussion

In recent years, with the advance of next-generation sequencing technology, mutated driver genes have been systematically identified for all major cancer types, especially through consortium projects such as TCGA or ICGC. But these studies usually characterize the mutational signals from patients with mixed genetic backgrounds or a single-patient population. A key next step to implement precision cancer medicine is to identify race-specific mutated drivers, which will lay a critical foundation for developing novel therapeutic strategies that target different patient populations.

Here we performed such an analysis of race-biased mutational features in ESCC patients by sequencing large patient cohorts and employing a rigorous analytic pipeline that explicitly considered various confounding factors. Compared to Caucasian patients, we identified one frequently mutated gene (*CSMD3*) with potential prognostic power and three race-biased mutated genes (*TP53*, *EP300,* and *NFE2L2*) in Asian patients. Additional efforts are needed to investigate their potential as biomarkers or therapeutic targets specific to Asian patients. The computational pipeline we developed can be readily applied to similar analyses for other cancers.

## Methods

### Sample collection

Human primary ESCC and corresponding adjacent non-tumor tissues (5 cm from the tumor site) were collected from patients who were diagnosed and received surgery as primary treatment at Fudan University Cancer Center (Shanghai, China) from September 2007 to June 2011. The tumor tissues were snap-frozen in liquid nitrogen immediately after surgical resection and then stored at −80 °C until they were analyzed. The clinicopathological features of the patients were collected from in-patient medical records. The pathological features were evaluated by independent pathologists according to the TNM staging system of the American Joint Committee on Cancer (AJCC 7th edition). All patients were followed up after primary treatment at intervals that increased from 3 months to 1 year until death. The study protocol was approved by the hospital ethics committee and informed consent was obtained from all participants.

### Whole-exome sequencing and targeted sequencing

Genomic DNA was extracted from the tissue specimens using QIAamp DNA kit (Qiagen). The libraries were then prepared using protocols recommended by Illumina. Briefly, 1 μg DNA was sheared to short fragments (200–300 bp) using Covaris S220. DNA fragments were end repaired and an adenylate blocker was added at the 3′ ends. Adaptors with barcode sequences were then ligated to both ends of the fragments. E-Gel was then used to select DNA fragments of the targeted size. Afterward, 10 cycles of polymerase chain reaction (PCR) were performed, and the resulting mixture was purified. Whole-exome capture was performed using the TruSeqExome Enrichment kit (Illumina) according to the manufacturer’s protocol, with minor modifications. After the libraries were amplified with 10 cycles of PCR, the capture probes were added and incubated for 24 h at 65 °C. The hybridized mixtures were then amplified with another 10 cycles of PCR. Validated DNA libraries were then sequenced on the Illumina Sequencing System (IlluminaHiSeq 2500). We included 283 “cancer-related genes” in the target enrichment panel as previously described^18^. Briefly, these genes included those recurrently mutated genes in gallbladder carcinoma, high-priority genes in Catalogue of Somatic Mutations in Cancer (COSMIC, http://cancer.sanger.ac.uk/cosmic/), genes related to drug sensitivity and highly mutated genes in gastrointestinal cancer. Targeted gene enrichment was performed with the TruSeq Custom Enrichment kits (Illumina).

### Sequencing data processing and mutation calling

Read pairs (FASTQ format) were trimmed and filtered with fastq-mcf (https://github.com/ExpressionAnalysis/ea-utils). The resulting high-quality reads were aligned to the human reference genome (GRCh37) using Burrows-Wheeler Aligner (BWA 0.7.12)^26^. BAM files were processed by Genome Analysis Toolkit^27^ to improve alignment accuracy. Major steps included marking duplicates, local realignment around high-confidence insertion and deletions and base quality recalibration. We then used several popular callers, including Muse^28^, MuTect2^29^, SomaticSniper^30^, Radia^31^, and VarScan2^32^, to identify somatic point mutations. Only mutations reported by at least two callers were used in further analyses. Low coverage and strand-biased mutations were filtered out. To further reduce false positives and miscalled germline events, we used MuTect2 to call point mutations on all the normal samples. Any of these germline mutations, if found in more than one normal sample, were removed from our final list of somatic mutations. To assess the accuracy of our mutation calls, we obtained TCGA MC3 mutation data from Synapse (syn5917256, version 0.2.8) and calculated the (median) fraction of MC3 non-silent mutations (e.g., missense, nonsense, and nonstop) called in our mutation set across the same set of TCGA samples and vice versa. To further validate our somatic mutation calls, we performed targeted sequencing on the same Chinese samples (*n* = 75). For all somatic mutations called from WES data, 592 mutations positions had a depth of coverage ≥200 in the targeted sequencing data. Among them, 564 somatic mutations were also detected in the targeted sequencing data, resulting in a true positive validation rate of 95.3%.

To remove the confounding effects due to different sequencing coverages, we implemented a random read downsampling strategy to achieve similar sequencing depths (<10% standardized difference) for each exon across the three patient populations. We repeated downsampling for 10 times and called somatic mutations independently and used consensus mutations (those called in ≥8 times) for further analyses.

### Clustering of patient samples by common SNP status

For all TCGA and Chinese samples, we obtained the read coverage information of exonic common SNPs in the dbSNP (build 147) using bam-readcount (https://github.com/genome/bam-readcount) for each normal sample. The minimum base quality was set to 15, and our analysis included 168,275 common SNPs with the coverage of ≥8 in each sample. We recoded the genotypes into 0, 1, 2 based on the wild-type/heterozygous/homozygous status of the SNPs. The WES samples were hierarchically clustered by the “dendextend” package in R^33^.

### Bioinformatic analysis on mutation data

To identify significantly mutated genes, somatic mutations were annotated using Oncotator^34^. MutSigCV (V1.4)^19^ was applied to identify significantly mutated genes with default covariate tables. Genes with *q* (FDR) < 0.1 were considered to be significantly mutated. We performed survival analyses using the “survival” package in R^35^. Kaplan–Meier survival analysis curves and the univariate Cox regression model were used to test for survival differences between groups (capped at 3 years). We tested for the mutual exclusivity between two patient populations using CoMEt^36^, which uses a Markov chain Monte Carlo algorithm to compute the marginal probability of observing pairs of alterations. We used Genotype Query Tools^37^ to calculate the fixation index for each SNP site reported by the 1000 Genomes Project (phase 3) between southern Han Chinese (CHS) and the European super population (EUR). The SNP status of rs113671272 in Chinese and Vietnamese patients with WES was inferred from off-target reads with a minimum of 3 read coverage. To examine the SNP effects on the gene expression, we obtained the genotypes of rs113671272 in TCGA samples from ICGC whole-genome sequencing data, and obtained the mRNA expression level of *NFE2L2* (based on the longest transcript uc002uli.3) from Fire Browser (http://firebrowse.org, version 2016_01_28). Integrating both genotype and expression data, our analysis included 12 cancer types in which each comparison group (samples with or without the SNP) contained at least three samples, and the SNP effect on gene expression was assessed by paired Wilcoxon rank-sum test. The cancer samples with *NFE2L2* somatic mutations were excluded from the analysis.

### Propensity score adjustment

We collected and obtained clinical characteristics (age at diagnosis, gender, tumor stage, smoking history, and alcohol consumption history) for all ESCC samples. We then employed a propensity score analysis to identify genes that preferentially showed higher mutation frequencies in one race group. For a comparison between Asian and Caucasian patient groups, we first calculated the propensity score using logistic regression with “race” as the responsible variable. We used the matching weight scheme^16^ continuously to assign weights for each sample based on the propensity scores to search for balance. When the standardized difference of the weighted propensity scores between two race groups was smaller than 10%, we considered the clinical characteristics balanced between the propensity score weighted samples. We then compared gene mutation frequencies between the two race groups by supplying the weights to a weighted chi-squared test, and calculated *P*-values and FDRs. Any gene with FDR ≤ 0.1 was considered biased in the two groups. We confirmed the statistical significance by randomly shuffling the race labels of the samples and repeated the above procedures 100 times. We calculated the statistical significance by comparing the number of significant features calculated from our real data to those from the permutated data.

### Data availability

The WES and targeted-sequencing data of Chinese ESCC samples have been deposited in the NCBI Sequence Read Archive (SRA) under Bioproject (accession number: PRJNA399748). The WES data of TCGA ESCC samples are available from NCI Genomic Data Commons (https://portal.gdc.cancer.gov/). All relevant data sets for this study are available from the authors.

## Electronic supplementary material

Supplementary Information

Description of Additional Supplementary Files

Supplementary Data 1
