# Supplementary material for: Comparative genomic analysis of esophageal squamous cell carcinoma between Asian and Caucasian patient populations
Source: Nat Commun. 2017 Nov 16;8:1533. doi: 10.1038/s41467-017-01730-x (PMC5688099; doi:10.1038/s41467-017-01730-x)
Supplement: Supplementary file 1 — Supplementary Information [file 41467_2017_1730_MOESM1_ESM.pdf]

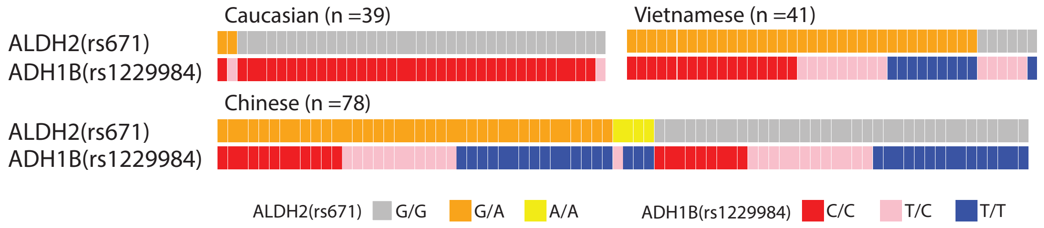

Supplementary Figure 1. Polymorphisms in alcohol metabolism genes. Non-synonymous SNPs in aldehyde dehydrogenase (ALDH2) and alcohol dehydrogenase (ADH1B) were more frequent in the Vietnamese and Chinese cases.

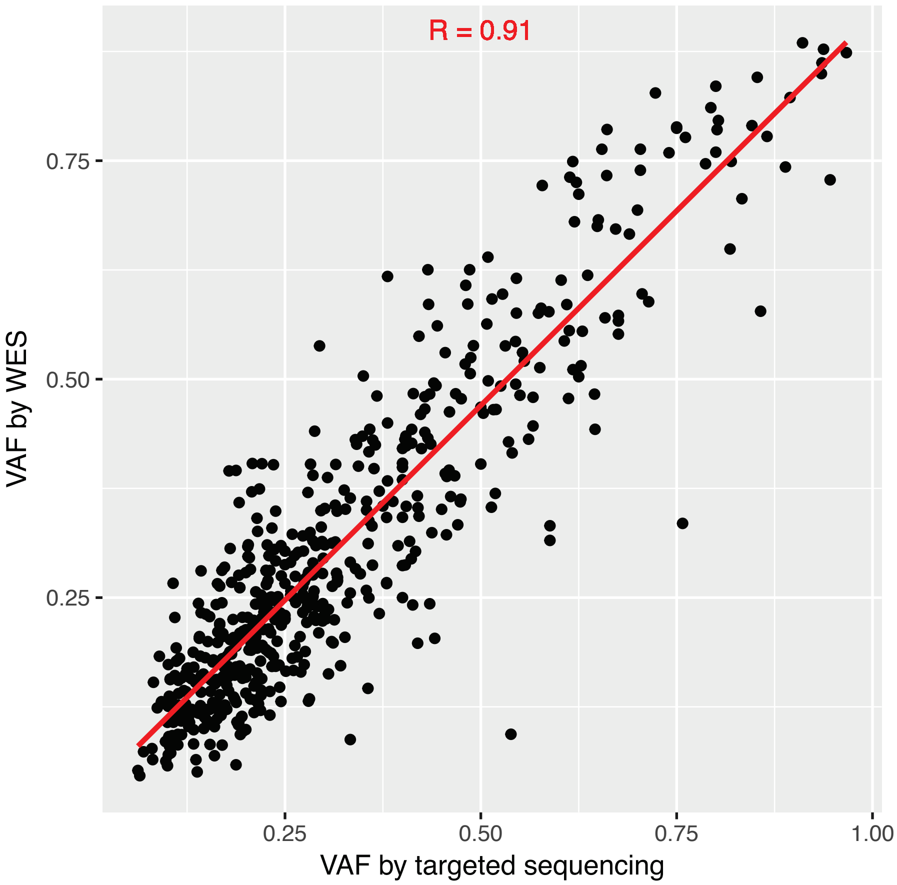

Supplementary Figure 2. The correlation of variant allele frequency between whole exome sequencing and targeted sequencing. Each point in the figure was a somatic SNV with high coverage (>=200X) in targeted sequencing samples. A total of 592 SNVs were plotted. Pearson correlation was 0.91.

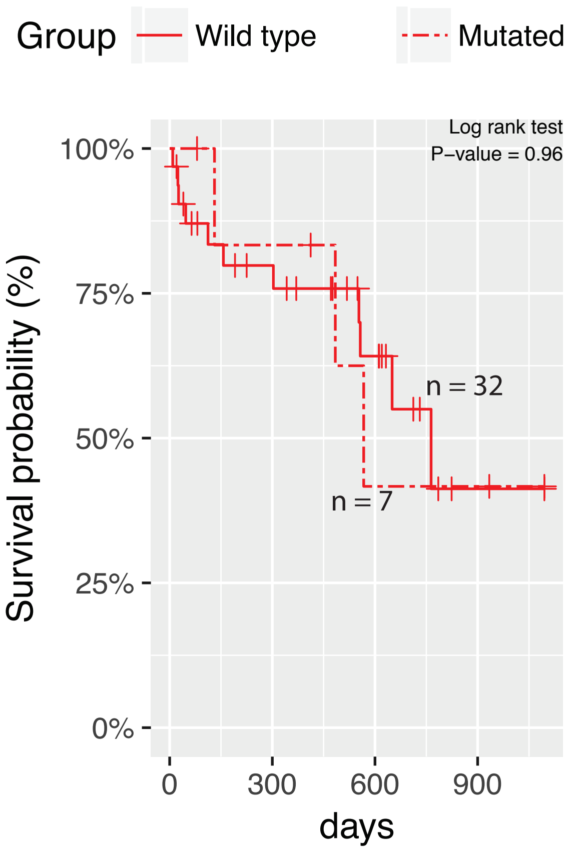

Supplementary Figure 3. 3-year survival rates of Caucasian cohort grouped by CSMD3 somatic mutation status. Log rank test p value was 0.96. Mutated cases were shown in dashed lines and wild type samples were shown in solid lines.

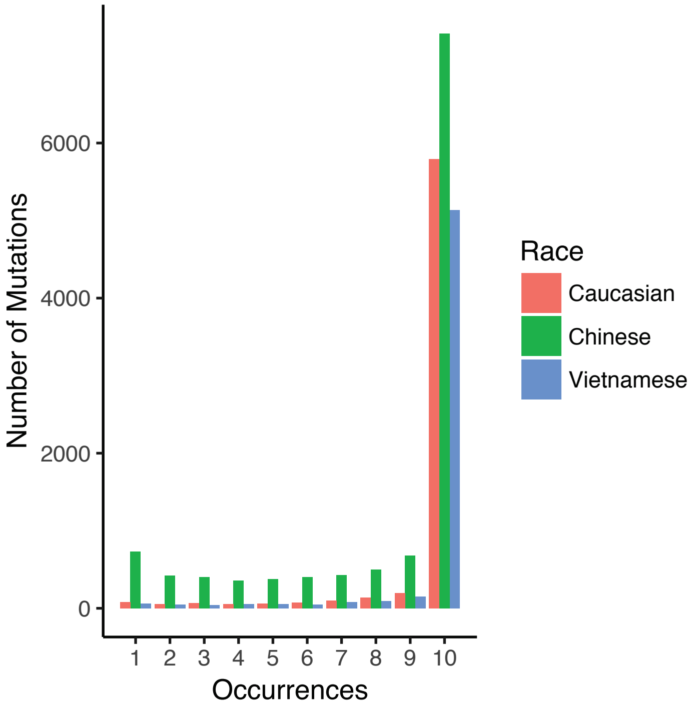

Supplementary Figure 4. Summary of exonic mutations from 10-times down-sampling and mutation calling. The x-axis is the number of times each mutation could be identified. The y-axis is the number of somatic mutations in each occurrence category.

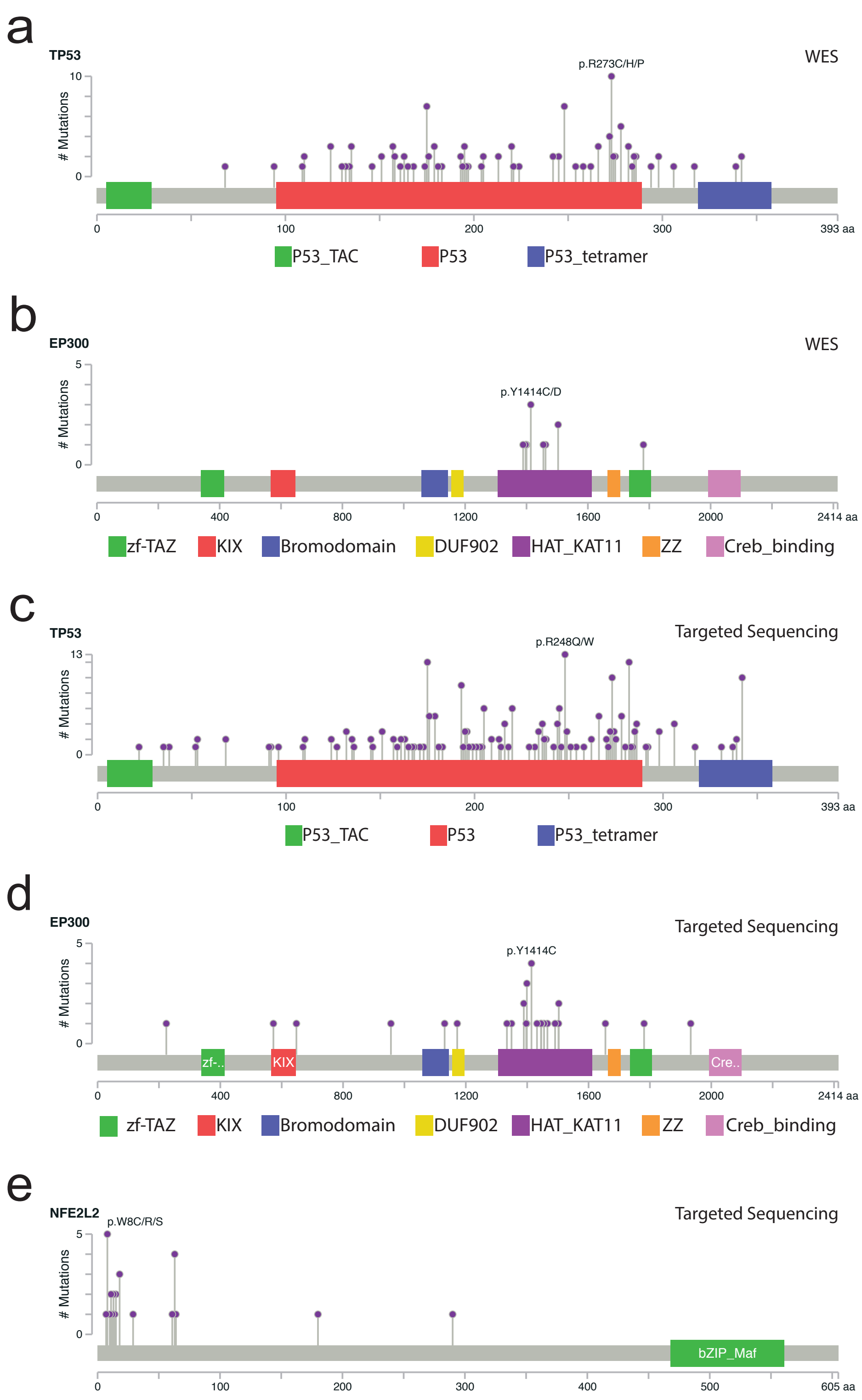

Supplementary Figure 5. Schematics of protein changes in race-biased gene products. (a), (b) are for WES cases. (c), (d), (e) are for chinese targeted sequencing cases. (a), (c) Scattered distribution of somatic mutations in TP53. (b), (d) Clustered mutations within the HAT_KAT11 domain of NFE2L2. (e) Two clustered mutation hotspots on NFE2L2.
