# Supplementary material for: Comparative genomic analysis of esophageal squamous cell carcinoma between Asian and Caucasian patient populations
Source: Nat Commun. 2017 Nov 16;8:1533. doi: 10.1038/s41467-017-01730-x (PMC5688099; doi:10.1038/s41467-017-01730-x)
Supplement: Supplementary file 2 — Description of Additional Supplementary Files [file 41467_2017_1730_MOESM2_ESM.docx]

**Description of Additional Supplementary Files**

File Name: Supplementary Data 1

Description: Clinical characteristics of Chinese individuals with ESCC in this study.
